# Demography and the dual epidemics of tuberculosis and HIV: Analysis of cross-sectional data from Sub-Saharan Africa

**DOI:** 10.1371/journal.pone.0191387

**Published:** 2018-09-07

**Authors:** Gambo Aliyu, Samer S. El-Kamary, Alash’le Abimiku, William Blattner, Manhattan Charurat

**Affiliations:** 1 Institute of Human Virology, University of Maryland School of Medicine, Baltimore, Maryland; 2 Department of Epidemiology and Public Health, University of Maryland School of Medicine, Baltimore, Maryland; Yale University Yale School of Public Health, UNITED STATES

## Abstract

**Background:**

Convergence of tuberculosis (TB) and HIV epidemics is associated with higher morbidity and mortality risks and understanding their distribution across key demographic factors is essential for prevention and control. This analysis examines the prevalence of TB, HIV and TB-HIV coinfection across age and gender in patients with presumptive TB seeking care at the National TB and Leprosy Training Center in Nigeria.

**Methods:**

Samples from 1603 presumptive pulmonary TB cases who provided informed consent were evaluated with a sequential testing algorithm that included a smear microscopy, cultures in liquid and broth media and then genotyping by Hain line probe assays. HIV was serially tested with two HIV rapid assays and retested with a third assay in non-conclusive samples.

**Results:**

Twenty-three percent (375/1603) had confirmed pulmonary TB infection, 23.6% (378/1603) were positive for HIV infection and 26.9% (101/375) of the confirmed TB cases were HIV co-infected. Males had a higher prevalence of TB: 27.6% vs. 18.0%, p < .0001; and a lower prevalence of HIV: 19.0% vs. 29.6%, p < .0001. In the age range of 25–29 years, males were twice as likely to have TB (OR = 2.2; 95% confidence interval [CI]: 1.3–3.9, p = 0.0032) while females were five times more likely to have HIV (OR = 4.8; 95% CI: 2.6–8.9, p < .0001). Persons with TB-HIV coinfection were more likely to be young, female and less likely to be married.

**Conclusion:**

Younger females with a high burden of HIV may be under-diagnosed and under-reported for TB in Nigeria. Community programs for intensified and early detection of TB and HIV targeting younger females are needed in this setting.

## Introduction

Age, biology and behavior interact to influence transmission and distribution of infectious diseases such as tuberculosis (TB) and HIV.[1–9] Globally, more adult men than women are diagnosed with pulmonary TB and die from the disease in different regions of the world particularly among HIV negative persons.[10–14] The reasons for increased susceptibility of men to pulmonary TB may be due to a combination of biological and behavioral differences that make disease acquisition and reporting higher in men than in women. Gender specific barriers for seeking healthcare, access to care, diagnosis, treatment and adherence may also contribute to a higher rate of detection and notification of TB in men compared to women, especially in low income countries.[13, 15–19]

In the early phases of the HIV epidemic, the disease burden was largely concentrated among male homosexuals[20–23] and intravenous drug users[24–26]. However, as the epidemic gradually expanded and became widely transmitted heterosexually, the increased vulnerability of women due to biology[27], behavioral risks[28] and gender inequality[29], resulted in an increased burden in females[30]. The combined effects of poverty[31], HIV infection and the tendency of women to be the primary care provider for TB affected members of the family[32] resulted in a greater incidence of TB infection and mortality among women of reproductive age in Africa, ultimately accounting for 90% of the global HIV associated TB mortality in women.[33]

The age distribution of HIV and TB infections is significantly different for males and females. Before the emergence of the HIV epidemic, young females (15 to 34 years) in industrialized countries had higher TB rates than males of the same age. HIV on the other hand is disproportionately reported among young females, presumably due to greater awareness and testing, and this is more evident in Sub-Saharan Africa where in the young age group of 15–24 years, three HIV positive women are reported for every HIV positive man.[34] Understanding the correlation between age, gender and the distribution of TB and HIV are important for prevention and control. The objective of this study is to provide insight into age-gender differences in the distribution of TB and HIV among presumptive TB patients in Nigeria.

## Materials and methods

Participants were enrolled into this cross-sectional study from two TB clinics in the state of Kaduna, Nigeria: the National TB and Leprosy Training Center (NTBLTC), Zaria, and at the Barau Dikko Hospital (BDH), in Kaduna City [35]. Kaduna is located at 10.52° North latitude, and 7.44° East longitude. It is situated some 614 meters above the sea level with an estimated population of 1,582,102. The NTBLTC is the largest TB referral center in northern Nigeria, while the BDH located in the city of Kaduna, is the major referral center within the state. Several hundred patients receive TB/HIV treatment at these facilities. Inclusion criteria included suspected cases with symptoms suggestive of TB and unknown HIV status visiting the facilities for the first time were enrolled. Participants had to be 18 years or older and provided written informed consent. For each patient, a supervised spot sputum sample was collected in the clinic, and HIV status was determined. To obtain an unbiased estimate of HIV among patients presenting with TB symptoms, we excluded known HIV-infected cases referred from the HIV clinic. Detailed description of the population and enrollment into this study, including the ethical challenges, were previously described [35, 36]. The protocol for this study was reviewed and approved by the University of Maryland’s Institutional Review Board and the Nigerian Health Research Ethics Committee. The initial sample size for this study was estimated based on the expected proportion of TB cases in the study population that would be due to *Mycobacterium bovis*. We used a 95% confidence interval with a desired margin of error within 3% of the true proportion and a previously reported *Mycobacterium bovis* prevalence of 15% to arrive at 544 as the number of confirmed TB cases required to estimate the prevalence.

The assays were conducted at the TB reference laboratory established by the Institute of Human Virology, Nigeria (IHVN) at the NTBLTC, Zaria. Sputum samples from presumptive TB patients were evaluated with a testing algorithm that included sputum smear microscopy, cultures in liquid and broth media and then characterized with the Genotype MTBC (Hain) assay [37] Each patient provided three sputum samples: (1) on the ‘spot’ (first day), (2) an early morning sputum sample collected at home before coming to the clinic the next day; and (3) on the ‘spot’ upon return the next day. The smears were then stained with the Ziehl-Nielsen (ZN) technique [37] Examination of the slides was done under 100x microscope magnification. For the purpose of this study, smears were graded as positive when five or more bacilli were detected in at least 100 fields.

### Mycobacterial culture and isolates characterization

The early morning smear positive and smear negative samples were cultured in Mycobacterium Growth Indicator Tubes (MGIT) as previously described.[37]. Samples that flagged positive were removed from the machine and inoculated on Blood Agar to check for contamination. Then, a Ziehl-Neelsen (ZN) stain was performed to check for the presence of AFB followed by an SD-Bioline rapid test for the rapid identification of M. tuberculosis complex (TB). Samples that failed to show any growth after 42 days of incubation in the machine were removed and classified as negatives. Samples were considered positive for TB if in addition to the positive growth on the MGIT, they showed the presence of AFB on ZN stain and tested positive on the SD-Bioline.

**Fig 1:**
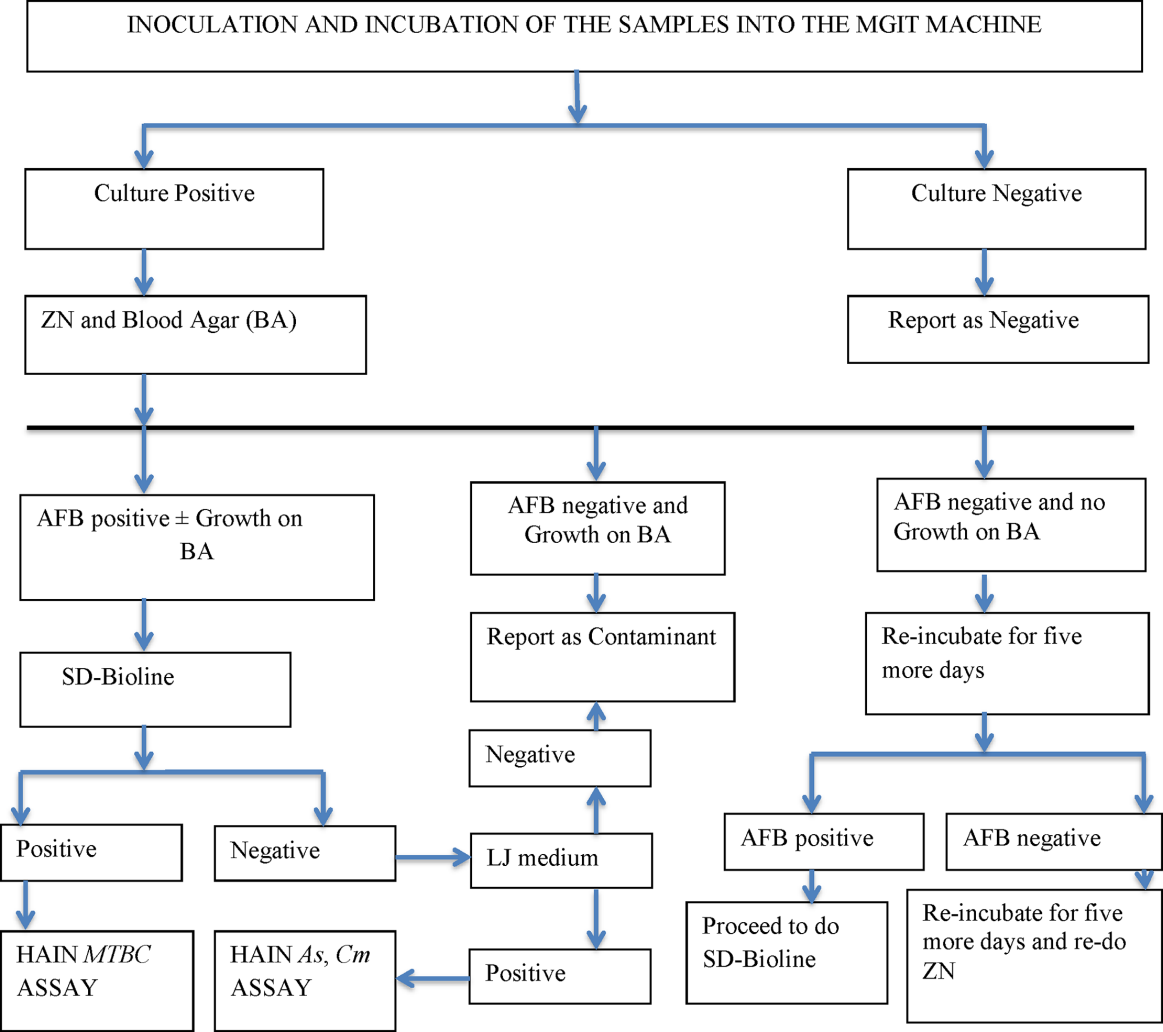
Flow chart for mycobacterial isolates detection and characterization. A flow chart illustrating the steps taken to identify and characterize mycobacterial isolates. ZN: Ziehl Neelsen Stain AFB: Acid Fast Bacilli SD-Bioline: Standard Diagnostic Bioline LJ: Lowenstein Johnson MTBC: Mycobacterium Tuberculosis Complex CM: Common Mycobacteria AS: Additional Species.

The culture confirmed TB positives were then characterized with Genotype MTBC. Samples positive for AFB on the ZN stain and negative for Mycobacterium TB complex on the SD-Bioline were further sub-cultured on Lowenstein Johnson (LJ) medium for a maximum of 42 days. A positive growth on LJ was followed by Genotype CM and AS Hain line probe assays (Hains Life science, Nehren, Germany) to isolate and characterize non-tuberculous mycobacterium present in the culture as outlined in Fig 1 and previously reported.[37]

### HIV antibody detection

An HIV testing algorithm in use by the President’s Emergency Plan for AIDS Relief (PEPFAR) program to detect HIV infection in Nigeria was applied in this study. Blood was serially tested with two HIV rapid assays (Trinity Biotech Uni-Gold™ (Co Wicklow, Ireland) and Abbott Determine® (Illinois, USA)). To consider a sample positive for HIV, detection of HIV antibody in both is required. If one test was not conclusive, then a third tie-breaker assay, Chembio STAT-PAK® (New York, USA) diagnostic assay was used. A positive test with the third assay confirmed positive HIV status; otherwise, the case was regarded as HIV negative.

### Data analysis

Data from the survey, medical records and laboratory tests were captured into different tables in a Microsoft Access database and applicable error checking keys were enforced at the start of the entry. Statistical analysis software (SAS, version 9.2) was used in the analysis. Missing values and outliers were detected and relevant corrections effected. Frequencies and proportions were computed for the categorical variables. Associations between categorical variables were evaluated with Pearson’s Chi-square or Fisher’s exact test. T-test was used to evaluate differences in the mean between the population groups. P-values of 0.05 or less were considered statistically significant and all probabilities were two-sided. Effect measures were adjusted by logistic regression analysis in which Odds ratio and 95% confidence intervals were reported.

## Results and discussion

A total of 1,657 patients presented to both clinics, and of those 1,603 (96.7%) subjects provided informed consent to participate in the study. There were no missing data for the key variables of age, gender, TB and HIV. The median age of the study population was 35 years, inter-quartile range (IQR) = 18. The prevalence of TB, HIV, and TB-HIV coinfection was 23.4% (375/1603), 23.6% (378/1603) and 6.3% (101/1603) respectively. Among the TB cases, 26.9% (101/375) were TB-HIV coinfected. The majority of participants were males, 901 (56.2%), and the TB prevalence was higher compared to females (27.6% vs. 18.0%, p < .0001) while the converse was true for HIV: 29.6% vs 19.0%, p < .0001 (females vs. males). The differences between males and females were statistically significant across other demographic and behavioral characteristics evaluated (Table 1).

**Table 1:** Demographic and behavioral characteristic by gender among presumptive TB cases.

**Demographic characteristics**		**Male N = 901**		**Female N = 702**		
		n	%	n	%	P-value
Age	<35 years	409	45.4	385	54.8	.0002
	35+ years	492	54.6	317	45.2	
Tuberculosis	Yes	249	27.6	126	18.0	< .0001
	No	652	72.4	576	82.1	
HIV infection	Yes	170	18.9	208	29.6	< .0001
	No.	731	81.1	494	70.4	
Cigarette smoking	Ever smoker	269	29.9	48	6.8	< .0001
	Never smoker	632	70.1	654	93.2	
Alcohol intake	Yes	157	17.4	54	7.7	< .0001
	No	743	82.6	648	92.3	
Education	Primary	497	55.2	484	69.0	< .0001
	Above primary	404	44.8	218	31.1	
Ethnicity	Hausa-Fulani	745	82.9	522	74.4	< .0001
	Other	156	17.3	180	25.6	
Marital status	Unmarried	283	31.4	206	29.3	.3730
	Married	618	68.6	496	70.7	
Setting	Rural	800	88.9	591	84.2	.0058
	Urban	100	11.1	111	15.8	

When the analysis was stratified by age, gender and absolute counts of infection type; differences in disease burden were larger in the younger age group of 25–29 years. Males had a higher burden of TB monoinfection compared to females in this age group: 17.5% vs. 5.5% but had a lower burden of HIV monoinfection: 3.6% vs 13.4% and TB-HIV coinfection: 6.9% vs. 11.9% compared to females (Table 2). Females in the 25–29 age group accounted for the highest proportion of persons with dual TB-HIV infection in the study sample analyzed. Tests of significance for these differences in the age group 25–29 years (not shown in Table) showed that males were twice as likely to have TB (OR = 2.2; 95% confidence interval [CI]: 1.3–3.9, p = 0.0032) while females were five times more likely to have HIV (OR = 4.8; 95% CI: 2.6–8.9, p < .0001) and twice as likely to have TB-HIV coinfection (OR = 2.1; 95% CI: 0.8–5.4, p = 0.13).

**Table 2:** Age, gender and the distribution of TB monoinfection, HIV monoinfection and TB-HIV coinfection among presumptive TB cases.

**Demography**		**TB+ HIV+** **N = 101**		**TB+** **N = 274**		**HIV+** **N = 277**		**TB- HIV-** **N = 951**	
**Age in years/***(n)*	Gender	n	%	n	%	n	%	n	%
<20 *(55)*	male	0	0.0	11	4.0	1	0.4	13	1.4
	female	0	0.0	11	4.0	4	1.4	15	1.6
20–24 *(209)*	male	7	6.9	32	11.7	3	1.1	64	6.7
	female	9	8.9	14	5.1	23	8.3	57	6.0
**25–29 *(271)***	**male**	**7**	**6.9**	**48**	**17.5**	**10**	**3.6**	**80**	**8.4**
	**female**	**12**	**11.9**	**15**	**5.5**	**37**	**13.4**	**62**	**6.5**
30–34 *(259)*	male	14	13.9	32	11.7	21	7.6	66	6.9
	female	12	11.9	14	5.1	29	10.5	71	7.5
35–39 *(209)*	male	13	12.9	20	7.3	27	9.8	71	7.5
	female	3	3.0	3	1.1	25	9.0	47	4.9
40–44 *(161)*	male	4	4.0	17	6.2	24	8.7	48	5.1
	female	5	5.0	7	2.6	19	6.7	37	3.9
45–49 *(108)*	male	4	4.0	5	1.8	16	5.8	32	3.4
	female	2	2.0	5	1.8	11	4.0	33	3.5
50–54 *(112)*	male	3	3.0	10	3.7	8	2.9	46	4.8
	female	2	2.0	4	1.5	6	2.2	33	3.5
55–59 *(66)*	male	2	2.0	3	1.1	3	1.1	31	3.3
	female	1	1.0	1	0.4	6	2.2	19	2.0
60–64 *(61)*	male	0	0.0	7	2.6	0	0.0	31	3.3
	female	0	0.0	4	1.5	1	0.4	18	1.9
65+ *(92)*	male	1	1.0	9	3.3	2	0.7	55	5.8
	female	0	0.0	2	0.7	1	0.4	22	2.3

**Fig 2:**
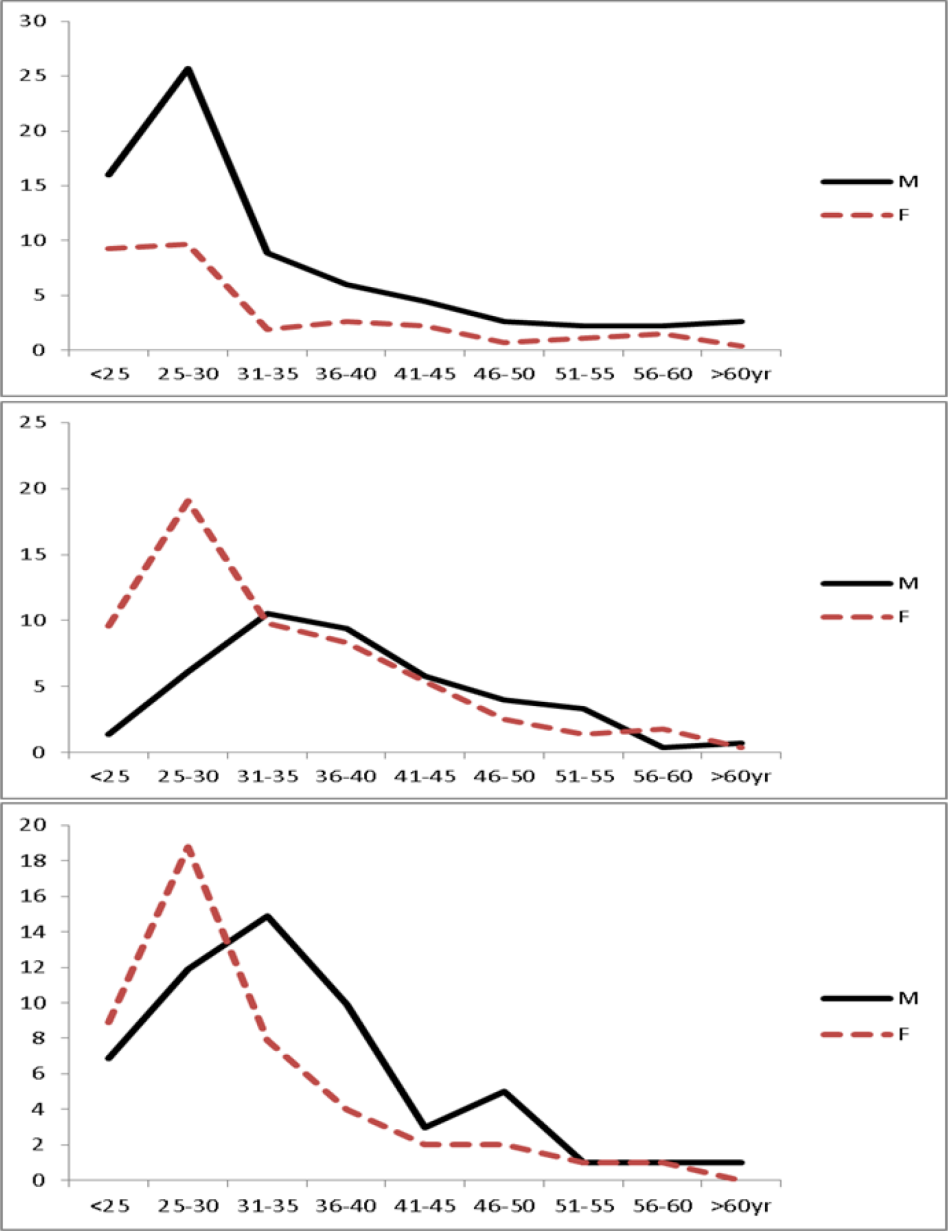
Variations in HIV, TB and TB-HIV prevalence across gender. M (dashed line) = males and F (solid line) = females; Top panel = HIV monoinfection; middle panel = TB monoinfection and bottom panel = TB-HIV coinfection.

After the age of 29 years, the gender difference in HIV and TB-HIV prevalence decreases with males contributing more to the burdens of HIV and TB-HIV infections in the age group 35–39 years, males vs. females: 9.8% vs. 9.0% for HIV and 12.9% vs. 3.0% for TB-HIV. The variations in TB, HIV and TB-HIV infections by gender across age groups are summarized in Fig 2.

Evaluating the correlates of TB-HIV coinfection in bivariate and multivariable logistic regression analyses, we found that the odds of TB-HIV coinfection after adjustment for educational status, ethnicity and settlement was significantly higher for unmarried patients and those with a positive history of cigarette smoking and alcohol consumption. The odds of TB-HIV coinfection were also higher for females compared to males although not statistically significant (Table 3).

**Table 3:** Multivariable logistic regression for risk of TB-HIV coinfection in presumptive TB cases.

**Variables**	**Unadjusted**		**Adjusted**	
	OR	95% CI	OR	95% CI
Gender				
Male	Ref			
Female	1.1	0.7–1.6	1.4	0.9–2.1
Age				
35+ years	Ref			
<35 years	1.5	1.2–1.8	1.4	0.9–2.3
Marital status				
Married	Ref			
Unmarried	2.0	1.3–3.0	1.6	1.0–2.5
Cigarette smoking				
Never smoke	Ref			
Ever smoke	2.2	1.4–3.4	1.9	1.1–3.1
Alcohol intake				
Never drink	Ref			
Ever drink	2.6	1.6–4.2	2.0	1.2–3.5

Adjusted for level of education, ethnicity and type of settlement

OR: Odds Ratio

Our findings show that the burden of TB and HIV are highest below the age of 30 years. Gender differences in the distributions of TB, HIV monoinfection and TB-HIV coinfection are also largest below the age of 30 years in this setting. The high occurrence of TB in the younger males with less burden of HIV compared to females of similar age group indicates a pattern of TB notification in males consistent with previous reports[14] and suggests a possible influence of environmental factors for the increased risk of TB infection among the males. Excessive exposure to seasonal dust among young males who dominate outdoors activities in northern Nigeria[37] and the gender gap in the ability to report and seek health services with higher tendencies in males than females[13, 38] may also contribute to the observed difference in notification of TB in favor of younger males.

The high prevalence of HIV monoinfection and TB/HIV coinfection in young females and the direction of the association between female gender and occurrence of TB and HIV towards statistical significance after adjusting for potential confounders could be a reflection of how these two diseases are now spreading rapidly among young females in this setting. It seems probable that TB prevalence among young females is largely driven by HIV. Notification of TB without associated HIV in this group is 16% lower compared to the young males. The findings underscore the importance of provider initiated HIV testing services (HTS) to all presumptive TB cases. The yield of TB-HIV coinfection and the magnitude of the difference across age and gender might have been under reported if HTS was offered only to patients after their TB status was confirmed.

Community awareness programs for TB and HIV in this setting should focus more on young women and girls. Access to HIV prevention programs should be extended to widows and single girls in addition to the cultural teachings that encourage staying chaste until marriage. At facility level, intensified case findings with emphasis on active clinical symptom screening for TB and the use of more sensitive diagnostic tools like the WHO-recommended GeneXpert in all HIV positive presumptive TB will increase TB-HIV notifications in this setting. Now that the landscape of TB and HIV epidemic control is strategically shifting towards ensuring that 90% of all people living with HIV will know their status, 90% of those who know their status will receive anti-retroviral therapy and of those, 90% will achieve viral suppression by 2020 (the UN 90-90-90 goals)[39–41], intensified and early diagnoses of TB and HIV will be of greater public health relevance if younger infected persons are promptly initiated on treatment and sustained until TB cure or successful completion of treatment, along with sustained HIV viral load suppression to below detection limit.

Given that over 80% of the presumptive TB cases enrolled in this study came from rural or semi-rural settings and the evidence showing strong correlation between HIV awareness creation and service utilization in rural Nigeria,[42] community awareness and sensitization may be used to increase uptake of TB and HIV services in this setting. However, there is the likelihood of younger persons not coming forward at early stages of stigmatized diseases like TB and HIV; innovative strategies at the community level may therefore be necessary. Efforts to partner with key opinion leaders like religious scholars and youth organizations including student unions should be intensified. While community effort pushes symptomatic young patients to facilities for screening, health workers at TB and HIV facilities should retain them in care by learning about, and addressing, their unique needs and challenges.

Our study has certain limitations, including the cross-sectional design which cannot determine temporal occurrences of TB and HIV in the coinfected subjects; our HIV testing algorithm might have missed cases of acute infections in discordant samples and the findings may only represent TB patients seen at facilities. However, we did not find any subject with discordant HIV tests and the facilities remain the source of primary data for global TB reports and decision making in Nigeria. The major strengths of this study are the large sample size, the utilization of a clearly defined TB testing algorithm that included testing of three separate sputum samples using smear microscopy, cultures in liquid and broth media followed by a genotyping assay; and for detecting HIV, the use of two rapid HIV tests and a third tie-breaker HIV assay if there was discordance between the first two HIV tests.

## Conclusion

The burden of TB and HIV infection in this setting are highest below the age of 30 years with younger males having higher rates of TB and less HIV infections, while younger females have higher rates of HIV and less TB infections. Integrated community programs for intensified and early detection of TB and HIV targeting younger females are needed in this setting.
